# Seasonal dynamics of mesozooplankton biomass over a sub‐Arctic continental shelf

**DOI:** 10.1002/ece3.7681

**Published:** 2021-05-25

**Authors:** Marc J. Silberberger, Paul E. Renaud, Ketil Eiane, Henning Reiss

**Affiliations:** ^1^ Institute of Oceanology Polish Academy of Sciences Sopot Poland; ^2^ Akvaplan‐niva Fram Centre for Climate and the Environment Tromsø Norway; ^3^ Faculty of Biosciences and Aquaculture Nord University Bodø Norway; ^4^ University Centre in Svalbard Longyearbyen Norway

**Keywords:** benthic invertebrate larvae, fish larval diet, length–weight relationship, Lofoten–Vesterålen region, marine food webs, meroplankton, pelagic‐benthic coupling, sub‐Arctic

## Abstract

Mesozooplankton research in high latitude ecosystems tends to focus on different life stages of *Calanus* spp. due to its biomass dominance and trophic roles. However, a complex seasonal succession of abundant smaller mesozooplankton taxa suggests that the ecological functioning of the mesozooplankton communities is more complicated. We studied the year‐round taxon‐specific biomass measurements and size distributions of mesozooplankton on a sub‐Arctic continental shelf based on formalin preserved samples. Our results confirm that *Calanus* spp. dominate the mesozooplankton biomass (81%). We show that commonly used length–weight relationships underestimate *Calanus* biomass in autumn and winter, and accordingly, a strong seasonal bias was introduced in our understanding of sub‐Arctic plankton communities. We observed two periods with considerable contribution of meroplankton, the planktonic larvae of benthic invertebrates, to the mesozooplankton biomass: (a) Cirripedia nauplii accounted for 17% of total biomass close to the coast in early April and (b) meroplankton comprised up to 12.7% of total biomass in late July. Based on these results, we suggest that meroplankton may play an ecologically important role in addition to their role in dispersal of benthic species. We conclude that the seasonal succession of the biomass of small‐sized holoplankton and meroplankton, often obscured by patterns in the *Calanus* biomass, should receive more attention as these smaller individuals are likely an important functional component of the pelagic food web.

## Graphical abstract

Mesozooplankton play a key role as the major trophic link between the pelagic primary producers and higher trophic levels. Here, we show that meroplankton, the planktonic larvae of benthic invertebrates, contribute considerably to the biomass of the smallest mesozooplankton size fraction (<1 mm) during spring, the period of first feeding for most major fish stocks spawning in the sub‐Arctic. We suggest that meroplankton plays an ecologically important role, contributing to early feeding of commercial fish species.

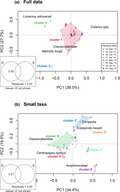

## 1 INTRODUCTION

Marine pelagic ecosystems sustain large populations of marine mammals, sea birds, and the majority of the large fish stocks. Within the pelagic food web, zooplankton play a key role as the major trophic link between the pelagic primary producers and higher trophic levels (Fenchel, 1988). In addition, their contribution to nutrient regeneration and dissolved organic carbon release supports the growth of phytoplankton and bacterioplankton (Banse, 1995).

After early detailed accounts of zooplankton community composition at high latitudes (*e.g*., Smidt, 1979; Wiborg, 1954), most zooplankton research today addresses macrozooplankton (krill, pelagic amphipods) or calanoid copepods of the genus *Calanus* (Orlova et al., 2015; Renaud et al., 2018). The latter is also the only mesozooplankton component considered in some models of ecosystem dynamics (Wassmann et al., 2006). The reason for this focus on a single genus is related to the dominance of *Calanus* spp. in mesozooplankton biomass during spring and summer (Aarflot et al., 2018; Arashkevich et al., 2002). The life cycle of *Calanus*, however, involves a descent away from surface waters to deep overwintering habitats from late summer to spring (Kaartvedt, 2000), while a diverse mesozooplankton community remains within the productive surface water throughout most of the year (Eiane et al., 2018; Silberberger et al., 2016). Consequently, a variety of planktonic groups may have an important ecosystem function (Hansen et al., 1999; Pasternak et al., 2008). Since many functional traits are linked to body size, mesozooplankton body size can be used as a proxy for their role in ecosystem functioning (Hébert et al., 2017; Litchman et al., 2013).

Small mesozooplankton (SMZ), such as small copepods and larval stages of planktonic and benthic invertebrates, are by far more abundant than *Calanus* throughout the year (Arashkevich et al., 2002; Pasternak et al., 2008). They are less studied than *Calanus*, partly due to methodological constraints and taxonomic uncertainty, though their importance as grazers has been recognized (Morales et al., 1991; Pasternak et al., 2008). Grazing impacts of SMZ on phytoplankton are directly comparable to that of the larger size fraction of the mesozooplankton community (Pasternak et al., 2008), and SMZ has been identified as an important component of the pelagic food webs in tidally mixed waters of the North Sea and the marginal ice‐zone of the Barents Sea (Pasternak et al., 2008; Williams et al., 1994). Meroplankton, larvae of benthic organisms, are even less studied than the holoplanktonic component of SMZ, and their potential ecological significance is mostly unknown. So far, most research on meroplankton focuses on their roles in dispersal and recruitment processes from a benthic perspective (Levin, 2006; Silberberger et al., 2016). However, meroplankton has been proposed to affect the pelagic ecosystem in various ways: (a) as grazers of autotrophic and heterotrophic micro‐ and nanoplankton in direct competition with holoplankton for resources (Pasternak et al., 2008; Turner et al., 2001), (b) as potential prey for higher trophic levels (Michelsen et al., 2017), or (c) through synchronized settlement that directs the assimilated carbon from the water column to the seafloor and reduces the grazing pressure on micro‐ and nanoplankton (Kirby et al., 2008; Sommer et al., 2000).

On the Lofoten–Vesterålen shelf, the zooplankton community was dominated year‐round by copepods (especially *Calanus*) between 1949 and 1950 (Wiborg, 1954). This study also reported that meroplankton rarely contributed with more than 10% to the total zooplankton abundance, with an annual average contribution of 2%–4%. While the abundance and the seasonal pattern of the numerically dominant holoplankton taxa and cod larvae was similar in 1949/1950 and 2013/2014 (Eiane et al., 2018; Wiborg, 1954), meroplankton has become a more abundant component of the zooplankton community (Silberberger et al., 2016). In 2013/2014, the annual average contribution of meroplankton to the mesozooplankton abundance was approximately 20% closer to the coast and around 5% over the central shelf (Eiane et al., 2018; Silberberger et al., 2016). Accordingly, a more important ecologic function of meroplankton in the Lofoten–Vesterålen region can be assumed. Whether this increased abundance of meroplankton is also reflected in the biomass, which is in most cases a more important measure for ecosystem functions, is still unknown.

To fully understand the functioning of pelagic ecosystems, knowledge about the seasonal and spatial distribution of zooplankton biomass and how it is divided among the various zooplankton components is indispensable. Zooplankton biomass‐spectra scale with important community processes, such as growth, mortality, or trophic structure (Hébert et al., 2017; Zhou et al., 2009), which have strong implications for the planktonic food web.

In addition to the lack of knowledge about the biomass associated with small and understudied taxa, virtually no study exists that measured the entire mesozooplankton community biomass on taxon level over space and time. In general, most studies use taxon‐ or stage‐specific individual weight data or length–weight relationships (*e.g*., Arashkevich et al., 2002; Aarflot et al., 2018). However, individual weight within a *Calanus* stage can vary by one order of magnitude (Aarflot et al., 2018) and different length–weight relationships can be found in the literature that differ by a factor of 5 for *Calanus* spp. (Cohen & Lough, 1981). No effort to correct for stage or size is normally made for smaller taxa. For *Oithona* spp., which is often considered the most abundant mesozooplankton taxon in the world's oceans (Gallienne & Robins, 2001), a fixed value of 0.003 mg/ind is used in studies of sub‐Arctic and Arctic mesozooplankton biomass (Blachowiak‐Samolyk et al., 2008; Mumm, 1991; Richter, 1994; Stübner et al., 2016). A similar approach has been applied for meroplankton, but with a lower taxonomic resolution. Typically, individual weights are applied for each meroplankton phylum, often ignoring individual growth stage or species identity. Consequently, it is likely that our understanding of seasonal and spatial variations of the mesozooplankton biomass in the sub‐Arctic is strongly biased, particularly for SMZ.

In this study, we follow the development of the mesozooplankton biomass on a sub‐Arctic open continental shelf over a 12‐month period. The main objectives were to (a) determine seasonal variation in the contribution of different mesozooplankton taxa to the total plankton biomass in the sub‐Arctic Lofoten–Vesterålen region; (b) identify seasonal and spatial patterns in biomass composition of small sized (<1 mm) mesozooplankton; (c) identify whether length–weight relationships can accurately estimate the seasonal development of mesozooplankton at the example of the year‐round abundant taxa (*Calanus* spp. and *Oithona* spp.). We hypothesize that meroplankton is an important, but so far underexplored, component of the mesozooplankton biomass in the sub‐Arctic that might have an important role in the functioning of the highly productive ecosystems in this region.

## 2 MATERIALS

### 2.1 Study region

**FIGURE 1:**
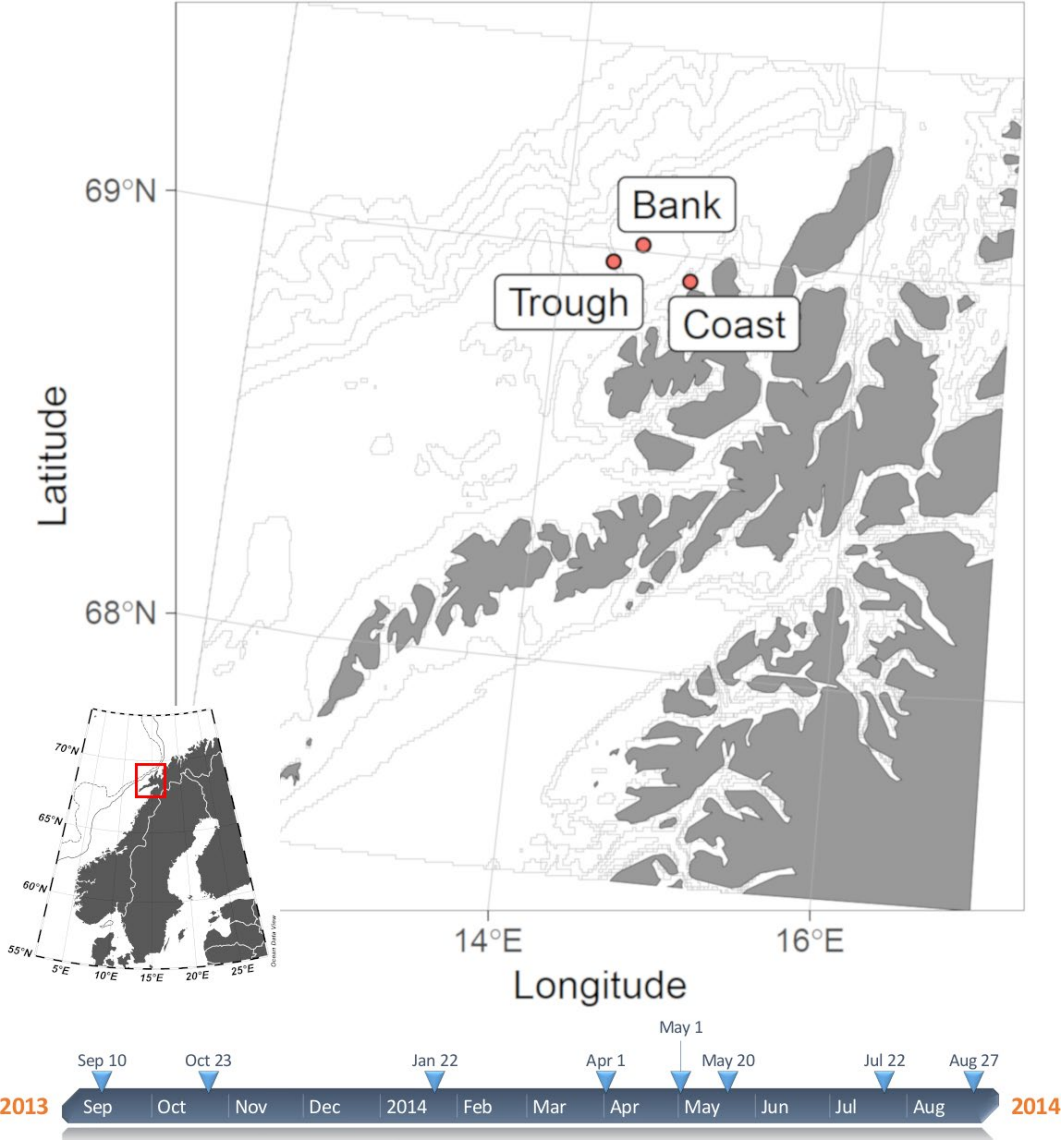
Map of the Lofoten–Vesterålen region. Sampling locations are indicated. Inset: Overview map of Scandinavia with position of study region indicated. Bottom: Timeline with sampling dates

Our study domain was the continental shelf off the coast of the Vesterålen islands, where the Norwegian shelf is at its narrowest (Figure 1). The Lofoten–Vesterålen region is among the most valuable marine regions in Europe, with a high importance for both the Barents Sea and the Norwegian Sea ecosystems (Olsen, 2009). It is an important fish spawning ground and larval drift area where first feeding fish larvae need to encounter suitable prey. The area is north of the Arctic Circle and is subject to strong seasonality in day length, temperature, and primary production (Eiane et al., 2018). Two major northward flowing currents, the Norwegian Atlantic Current (NAC) and the Norwegian Coastal Current (NCC), together with strong tidal forces, shape the hydrography of the region, causing an overall northward transport with retention of planktonic organisms over the shelf (Espinasse et al., 2017; Silberberger et al., 2016). The low salinity NCC overlies the high salinity water of the NAC like a wedge, with the greatest depth of the NCC layer along the coast. The strength, width, and depth of the NCC varies seasonally as it is partly driven by run‐off from land (Silberberger, 2017). Coastal regions, shallow banks, and cross‐shelf troughs represent the three landscape elements that characterize the region and differ with respect to depth and hydrography (Eiane et al., 2018; Silberberger et al., 2016). Water of the NCC (salinity <34.5) characterizes the relatively shallow coastal region (<50 m depth), which remains mixed for most time of the year. Further offshore, over banks (<100 m depth) and troughs (>200 m depth), a thermohaline vertical structure develops in early summer but typically weakens in late winter and early spring (Eiane et al., 2018). Below 100 m depth, the seasonal variability of salinity and temperature is much reduced compared to shallower waters.

### 2.2 Sampling

Plankton samples were collected at three stations during eight sampling events between September 2013 and August 2014 (Figure 1). Stations represent the landscape elements in the study region: Coast (C; depth: 40 m), bank (B; depth: 80 m), and trough (T; depth: 215 m). A WP2 closing net (opening = 0.25 m^2^, mesh size = 200 μm) sampled the complete water column in a single vertical tow at station C and B. At the deepest station (T), plankton was collected from two depth intervals: estimated maximum mixed‐layer depth (50−0 m) and deeper water (bottom−50 m). These samples were, however, combined for sample processing of this study. Samples were preserved in a buffered 4% formaldehyde‐seawater solution until further processing in winter 2017/18.

A detailed account of the holoplankton and meroplankton community in connection with environmental parameters collected during this field investigation is provided by Eiane et al. (2018) and Silberberger et al. (2016), respectively.

### 2.3 Sample processing

Depending on zooplankton density, samples were split to four, eight, or sixteen parts, depending on the concentration of organisms, with a Motoda plankton splitter. One part was diluted to 200 ml and used for the analysis. A fixed volume pipette was then used to collect 2 ml subsamples, and all individuals were identified under an Olympus SZX16 stereo microscope equipped with an Olympus SC180 digital camera that was connected to a computer with the image analysis software Olympus stream. Individuals of each taxon were transferred to 4 ml distilled water in preweighed aluminum weighing boats, which have been heated to 200°C for 3 hr prior to preweighing to remove any volatile residue and reach a stable mass. In addition, the length of each individual was measured (prosome length for copepods, longest extension of the body without appendages for all other taxa). Length measurements were made for up to 100 individuals of the most abundant taxa in a sample. For other taxa, length measurements were made for approximately 300–400 individuals per sample, which allowed to assess the length distribution of each taxon that was abundant enough for DW measurements. To ensure the establishment of unbiased length distributions, length measurements for a started 2 ml subsample were always completed. We chose the taxonomic resolution at the lowest level that allowed for a close to complete representation of the plankton community in the DW measurements (>95% individuals in each sample). If enough individuals of one taxon for dry weight measurement were collected, this taxon was disregarded in the remaining subsamples taken from that net haul. The weight of the collected plankton was determined after drying at 50°C for 18–26 hr (Stübner et al., 2016). Weighing boats were transferred to a desiccator to cool down to room temperature, before the mass was determined with a Mettler Toledo XS205 analytical balance with a precision of 0.01 mg. Temperature, humidity, and air pressure in the laboratory were monitored during preweighing and weighing to ensure accurate measurements.

To estimate our measurement error, three preweighed boats containing 4 ml of distilled water were dried as controls for each sample (i.e., 72 controls in total = 3 controls × 3 stations × 8 sampling dates). The average error was 0.016 mg, which was significantly different from 0 (*t*(71) = 12.269, *p* < .001). In comparison, the median weight gain of the containers with plankton was 0.2 mg (range: 0.01–26.46 mg).

### 2.4 Data processing

All biomass measurements were corrected by subtraction of the measurement error. Afterwards, all biomass values that did not exceed a weight gain of 4 standard deviations of the control samples (4 × *SD* = 0.043 mg) were discarded as unreliable (i.e., 25 of 223 measurements). These unreliable measurements were obtained when the sample size of uncommon species was small.

Preservation of mesozooplankton in formaldehyde may cause a biomass loss in the range from 37%‐43% depending on size and species (Giguère et al., 1989; Williams & Robins, 1982); thus, we assumed a loss of 40% for all taxa and corrected the measured biomass accordingly by multiplying by a factor of 1.67. Biomass loss during formalin preservation is typically large in the first weeks of preservation, after which it decreases gradually until a stable weight is reached after a few months (Schram et al., 1981; Wetzel et al., 2005). Since all samples were preserved for over three years, we assume that the biomass changes had stabilized. Finally, biomass estimates were standardized to 1‐m^2^ surface area by correcting for the used fraction of the total sample (split), the number of 2 ml subsamples used to collect the individual taxa, and the area sampled by the net (0.25 m^2^). Data were not corrected for tow depth, because zooplankton species are typically not distributed equally throughout the water column and occupy typically narrow depth ranges at any time (Eiane et al., 2018; Unstad & Tande, 1990). For comparison with volume‐specific data (per m^3^) in other studies, the presented values can be divided by the station depth. Biomass measurements per m^2^ surface area at the three sampling stations (C, B, T) in the ratio 1:2:5.4, therefore, indicate equal biomass per volume (averaged over the entire water column).

In addition, the average individual biomass for each taxon was calculated by dividing the corrected, but not standardized, biomass by the number of individuals that was collected for the measurement.

All reported results are based on biomass or average individual biomass per m^2^. Based on the biomass measurements, we used taxon‐specific conversion factors (Brey et al., 2010) to estimate organic carbon and energy content of the zooplankton community (Table 1).

**TABLE 1:** List of all taxa with at least one reliable dry weight (DW) measurement. The assigned size class (SMZ or LMZ) is given for each taxon. Applied conversion factors for the calculation of organic carbon content (mg C/mg DW) and energy content (J/mg DW) are given for each taxon together with the source taxa for the conversion factors

Taxon	Size class	mgC/mgDW	J/mgDW	Source taxa in Brey et al. (2010)
**Holoplankton**				
*Acartia longiremis*	SMZ	0.453	21.595	Median values for *Acartia* spp.
*Calanus* spp.	LMZ	0.502	26.889	Median values for *Calanus* spp.
*Centropages typicus*	SMZ	0.395	21.949	Median values for *C*. *typicus*
Cladocera	SMZ	0.439	17.680	Median values for marine Cladocera
Clausocalanidae	SMZ	0.497	21.222	Median values for Pseudocalanidae
*Clione* larvae	LMZ	0.300	17.355	Median values for *Clione* spp.
Copepoda nauplii	SMZ	0.396	24.080	Median values for marine Copepoda larvae
Euphausiacea larvae	LMZ	0.395	13.915	Median values for marine Euphausiacea larvae
*Fritillaria borealis*	SMZ	0.545	3.868	Median C/DW value for Appendicularia; Median J/mgDW value for marine swimming Tunicata
Hydrozoa	LMZ	0.101	7.864	Median values for marine swimming Hydrozoa
*Limacina retroversa*	LMZ^a^	0.333	15.955	Median values for *Limacina* spp.
*Metridia longa*	LMZ	0.510	28.549	Median values for *M. longa*
*Oikopleura* spp.	LMZ^a^	0.504	3.868	Median C/DW value for *Oikopleura* spp.; Median J/mgDW value for marine swimming Tunicata
*Oithona* spp.	SMZ	0.465	18.691	Median C/DW value for *Oithona* spp.; Median J/mgDW value for marine swimming Copepoda
*Paraeuchaeta* spp.	LMZ	0.587	25.100	Median C/DW value for *Paraeuchaeta* spp.; Median J/mgDW value for Euchaetidae
*Parasagitta elegans*	LMZ	0.399	17.366	Median values for *P. elegans*
*Temora longicornis*	SMZ	0.433	18.691	Median values for *T. longicornis*
**Meroplankton**				
Amphinomidae	SMZ	0.373	17.800	Median values for Polychaeta
Asteroidea	LMZ	0.130	9.944	Median values for Asteroidea
Bivalvia	SMZ	0.208	7.039	Median values for Bivalvia larvae
Bryozoa	SMZ	0.402	8.721	Median C/DW value for Animalia; Median J/mgDW value for Bryozoa
Cirripedia	SMZ	0.437	17.070	Median values for Cirripedia larvae
Gastropoda	SMZ	0.335	18.335	Median values for Gastropoda
Ophiuroidea	SMZ	0.142	5.425	Median C/DW value for Echinodermata; Median J/mgDW value for Ophiuroidea
Polychaeta	SMZ	0.373	17.800	Median values for Polychaeta
Decapoda zoea	LMZ	0.360	12.400	Median values for Decapoda larvae
**Ichthyoplankton**				
Fish eggs	LMZ	0.432	22.900	Median values for Teleostei larvae
Cod larvae	LMZ	0.432	22.900	Median values for Teleostei larvae

^a^ Taxa with a size range across both classes.

### 2.5 Data analysis

All multivariate statistical analyses were performed twice: once on the complete mesozooplankton biomass data, and once on a subset that included taxa smaller than 1 mm. This separate analysis of small taxa was performed for two reasons: (i) Body size is an important trait that has strong implications on food web structure and ecosystem functioning (Ye et al., 2013; Zhou et al., 2009), and (ii) only taxa smaller than 1 mm are commonly underrepresented in comparison with larger taxa in samples collected with a mesh size of 200 µm (Riccardi, 2010). Due to this sampling bias, the data for the smaller size fraction have to be considered nonquantitative and need to be interpreted with care.

The separation of the studied taxa in large and small was done according to their mean length (Table 1). *Limacina retroversa* and *Oikopleura* spp., however, displayed a large size variety between and within samples. Although their mean size was below 1 mm, we decided to exclude these two taxa from the small taxa for two reasons: (i) The size measurements of preserved samples (shell height of *L. retroversa* and head length of *Oikopleura* spp.) are underestimating the actual in situ size for both taxa. (ii) Some individuals were much larger than 1 mm, and we assume that our biomass measurements reflect primarily these big individuals. Since multiple individuals of one taxon from one sample were pooled to estimate biomass, we were not able to distinguish the weight of specific size classes of a single taxon and had to assign the entire biomass to the large or small size fraction. *Calanus* represented a third taxon that was represented by individuals in the small and large size fraction in our data. However, in contrast to the other two taxa, *Calanus* nauplii and copepodite stages were collected separately and accordingly the biomass associated with the different size fractions could be assigned correctly.

Each data set was subjected to a Hellinger transformation to make the data suitable for the Euclidean space (Legendre & Gallagher, 2001). The Hellinger transformation is defined as:

$$y_{\mathrm{ij}}^{\prime }=\sqrt{\frac{y_{\mathrm{ij}}}{y_{i+}}}$$

where *y_ij_* is the abundance of species *j* in sample *i,* and *y_i+_* is the total abundance in sample *i*. Principal component analysis (PCA) and hierarchical clustering were used to identify patterns in the biomass data. To select a suitable clustering method and identify meaningful clusters, we used an explorative approach following methods described by Borcard et al. (2018). Cophenetic correlations and Gower distance were used to select the clustering method that represented the Hellinger transformed data best. The evaluated clustering methods were single linkage, complete linkage, unweighted pair‐group method using arithmetic averages (UPGMA), and Ward's minimum variance clustering. Fusion level values, average silhouette widths, and matrix correlations between original dissimilarity matrix and binary matrices were used to identify the optimal numbers of clusters for the previously selected clustering method.

A species contribution analysis (SCA) was performed to identify taxa contributing to the differences among the identified clusters (van Son & Halvorsen, 2014).

Redundancy analysis (RDA) was used to partition the variation in the Hellinger transformed data on spatial and seasonal predictor variables (Borcard et al., 2018), thereby quantifying the spatial and seasonal component in the data. We used a factor variable, including the three sampling stations, as spatial predictor. We projected the sampling day on a circle by assigning sine and cosine of the day of the year to each sample. The resulting matrix was used as seasonal predictor in the variation partitioning. This circular predictor was chosen to ensure that samples from September 2013 and August 2014 were considered seasonally similar.

Three length–weight relationships for *Calanus* spp. and 4 length–weight relationships for *Oithona* spp. were applied to calculate individual DW for these two taxa (Table 2). We calculated the individual DW for each *Calanus* and *Oithona* with a prosome length measurement and consecutively used them to calculate the average individual DW in each sample. The calculated average individual DWs were then compared to the measured average individual DWs in each of our samples. Since neither formula for *Oithona* was developed for species in the north Atlantic or Arctic, the commonly used individual dry weight of 0.003 mg (Blachowiak‐Samolyk et al., 2008 and references therein) was also included in the comparison.

**TABLE 2:** Different length–weight relationships that have been applied to *Calanus* spp. and *Oithona* spp.

	*Calanus* spp.				*Oithona* spp.		
	Formula	Species	Region		Formula	Species	Region
(i)^a^	DW = 0.006458 × PL^3.9^	*C. finmarchicus*	North Atlantic and North Sea	(i)^d^	DW = 3	*Oithona* spp.	Svalbard
(ii)^b^	logDW = 0.735 × PL ‐ 2.5	*C. finmarchicus* + *C. glacialis*	Greenland Sea	(ii)^e^	logDW = 1.84 × logPL ‐ 4.84	*O. similis*	Inland Sea of Japan
(iii)^c^	DW =0.0084 × PL^3.4333^/0.9	*C. finmarchicus* + *C. glacialis*	Nansen Basin	(iii)^e^	logDW = 0.766 × logPL ‐ 2.20	*O. brevicornis*	Inland Sea of Japan
				(iv)^f^	DW = 3.405 × 10^–10^ PL^3.643^	*O. hebes*	Cananéia Lagoon (Brazil)
				(v)^f^	DW = 2.513 × 10^–11^ PL^4.113^	*O. oswaldocruzi*	Cananéia Lagoon (Brazil)

Note

Dry weight (DW) and prosome length (PL) in mg and mm for Calanus spp. and in µg and µm for Oithona spp., respectively. Original formula according to Mumm (1991) calculates ash free DW (AFDW) and was adjusted assuming an AFDW:DW ratio of 0.9 (Richter, 1994). References for each length–weight relationship indicated by superscript letters: ^a^Cohen and Lough (1981); ^b^Hirche (1991); ^c^Mumm (1991); ^d^Blachowiak‐Samolyk et al. (2008); ^e^Uye (1982); ^f^Ara (2001).

All statistical analyses were performed in R, version 3.5.0, making use of the vegan (Oksanen et al., 2018), veganUtils (Vihtakari, 2018), cluster (Maechler et al., 2018), and dendextend packages (Galili, 2015).

## 3 RESULTS

In total, we obtained biomass data for 28 taxa, representing 17 holoplankton and 9 meroplankton taxa, as well as two different developmental stages of ichthyoplankton (Table 1).

The relationships between measured biomass (mg DW/m^2^), calculated organic carbon (mg C_org_/m^2^), and energy content (J/m^2^) differed somewhat between the different plankton components. The C_org_:DW ratio and energy:DW ratio were higher in holoplankton and ichthyoplankton than in meroplankton, and thus, the contribution of meroplankton to the sample C_org_ and energy content was slightly lower than to the sample dry weight (Table 1, Appendix S1). However, this difference was small (maximum difference 4%, compare Appendix S1) and accordingly, general patterns of dry weight composition can be transferred to C_org_ or energy content.

### 3.1 Total biomass patterns

**FIGURE 2:**
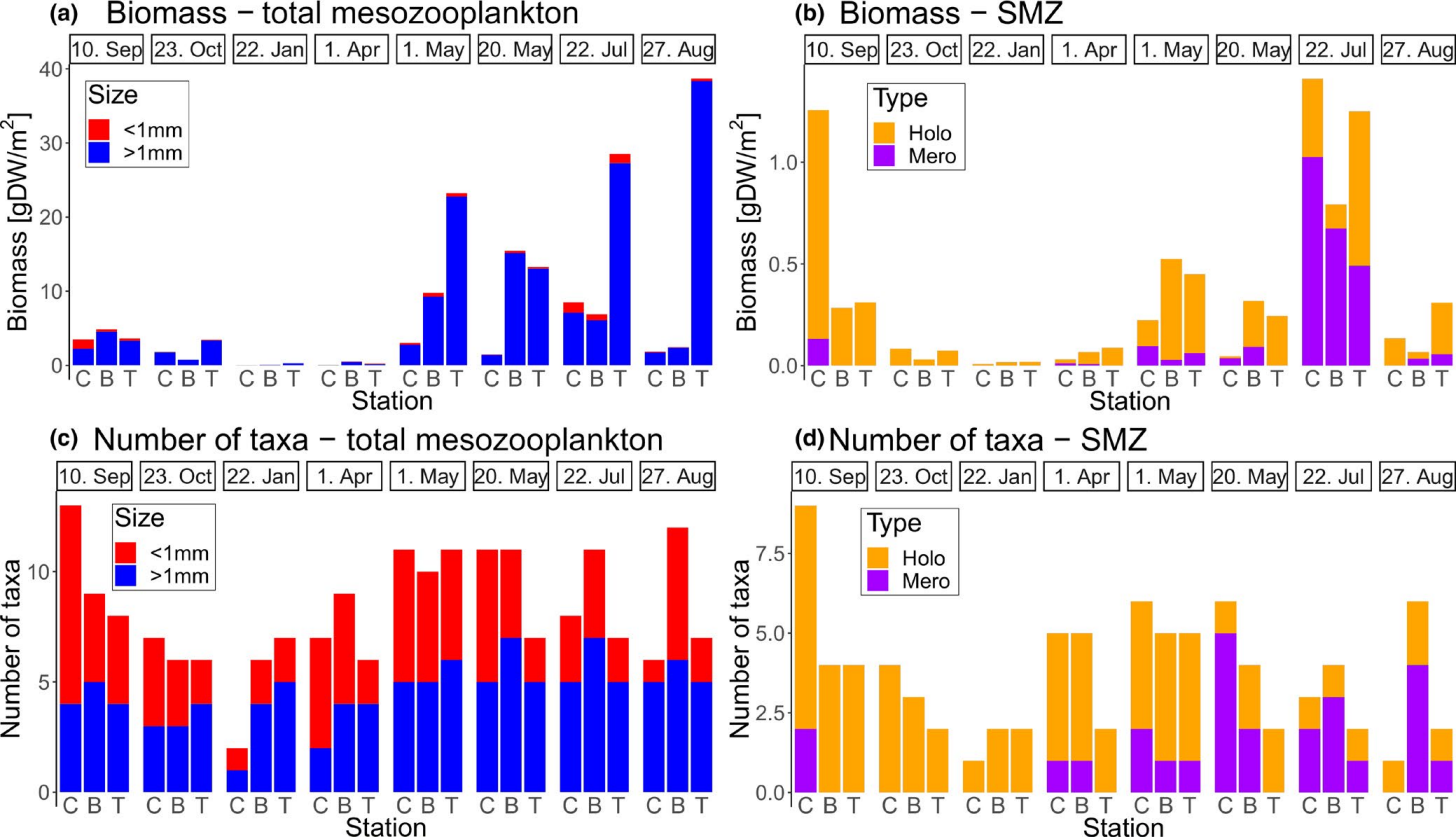
Barplots depicting total mesozooplankton biomass (a), total number of mesozooplankton taxa (b), biomass associated with small mesozooplankton (SMZ; <1 mm) (c), and number of SMZ taxa (d). Color indicates the fraction that was associated with taxa larger and smaller than 1 mm (a, b) or with holoplanktonic and meroplankton taxa (c, d). Sampling stations are abbreviated: C, Coast; B, Bank; T, Trough

**FIGURE 3:**
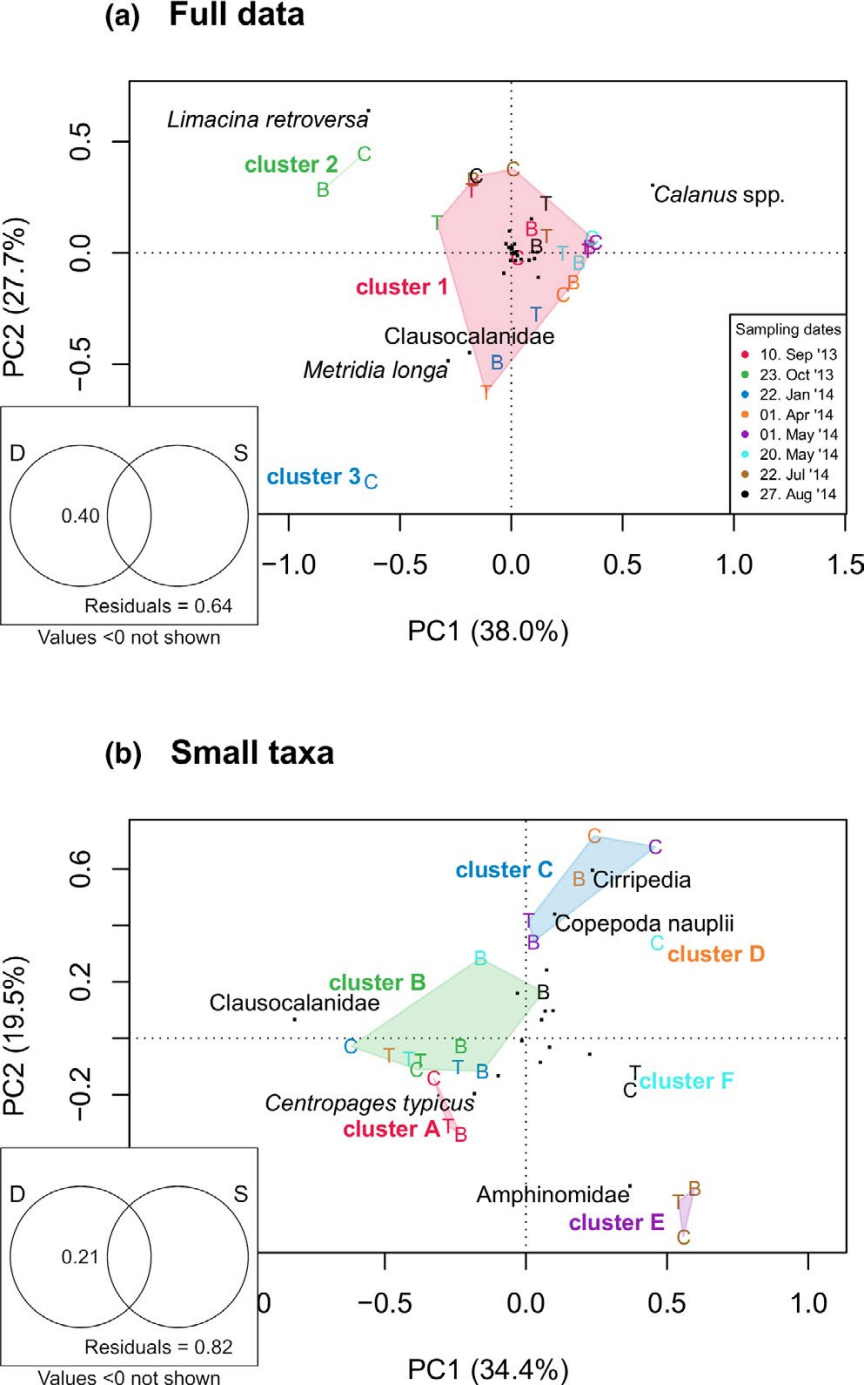
PCA ordination plots of the total mesozooplankton biomass (a) and the small taxa subset (b). Samples from sampling stations at coast, bank, and trough are indicated by the letters C, B, and T, respectively. Colors indicate sampling dates. Identified sample clusters are indicated. (Note: Cluster polygons for clusters containing only 1 or 2 samples are not visible.) Taxon loadings are indicated by black points, selected taxa are labelled. Venn diagrams display results of variation partitioning; date, D; station, S. (Note: The sum of the shown fractions exceeds 1, due to small negative adj. *R* ^2^ values for the fractions with no explanatory power. Such negative values are common in variation partitioning approaches and are interpreted as 0 (Legendre, 2008))

Averaged over the whole study period, a distinct spatial pattern with total low biomass near the coast (2,547 mg DW/m^2^), intermediate biomass over the bank (5,116 mg DW/m^2^), and highest biomass over the cross‐shelf trough (13,921 mg DW/m^2^) was observed. This pattern was caused by the holoplankton, which accounted for >90% of the total zooplankton biomass at all locations (Figure 2; Appendix S1). The meroplankton biomass component, however, showed an opposite pattern, with highest values at the coast (174 mg DW/m^2^), intermediate values over the bank (125 mg DW/m^2^), and lowest values at the deep station (76 mg DW/m^2^). Accordingly, the relative contribution of meroplankton to the total biomass differed from 6.8% at the coast‐near station to 2.6% and 0.5% over the bank and at the deep station, respectively. However, this was just an average trend and no consistent spatial pattern emerged across all sampling dates for biomass or the number of taxa that contributed to the biomass (Figure 2). Ichthyoplankton (mainly cod eggs and larvae) was present in samples from early April to mid‐May, with a biomass peak in early May. No consistent spatial pattern of ichthyoplankton distribution was observed throughout this period (Appendix S1). This general lack of a consistent spatial pattern was further confirmed by variation partitioning that showed station ID could not explain any variation in the complete data set (adj. *R* ^2^ = −0.04, Figure 3a).

Temporal succession of the plankton biomass followed a seasonal pattern with low values during winter and early spring (January–April), highest values in late spring and early summer (May–July), and intermediate values in late summer and autumn (August–October) (Figure 2a). A seasonal pattern was also confirmed by variation partitioning results, which indicated that the sampling date explained 40% of the variation in the data (Figure 3a).

**FIGURE 4:**
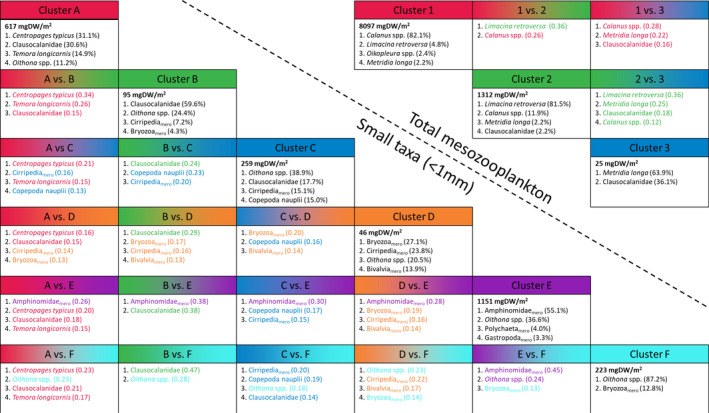
Results of the species contribution analysis (SCA) for the complete biomass data and the small taxa subset. The samples characterizing the different clusters are shown in Figure 3. The absolute plankton biomass and contribution (in %) of four taxa with the highest biomass are given for each cluster. Proportional contribution of taxa (given in decimals) to the difference between cluster pairs in the Hellinger transformed data is indicated for all taxa contributing at least twice the average contribution (complete data: 2 × 100% / 28 taxa = 7%; small taxa: 2 × 100% / 15 taxa = 13%). Meroplanktonic taxa are indicated

Overall, *Calanus* spp. dominated the zooplankton biomass on the Vesterålen shelf, accounting for 81% of the total biomass in this study (Figure 4). Furthermore, *Calanus* was the dominant taxon in 21 out of 24 samples. In the remaining three samples, the pteropod *Limacina retroversa* (St. C & B, 23.10.2013) and the copepod *Metridia longa* (St. C, 22.01.2014) dominated. Accordingly, PCA and hierarchical clustering identified three distinct clusters representing the samples of different species dominance (Figure 3a). Species contribution analysis (SCA) and species loadings of the PCA identified *Calanus* and *L. retroversa* as characteristic for clusters 1 and 2, respectively (Figure 3a, Figure 4). Station C in January (cluster 3), however, was rather characterized by the very low biomass of only two taxa that could be collected in necessary amounts to measure the biomass. This low number of taxa separated it clearly from all other samples that contained at least six taxa with sufficient biomass (Figure 2b).

### 3.2 Small mesozooplankton

The seasonal pattern of SMZ biomass development followed the general trend that was observed for the total mesozooplankton biomass (Figure 2). The on‐average elevated total biomass over the deep trough, however, was not reflected in the SMZ fraction. SMZ biomass was in general more equally distributed throughout the study area (Figure 2).

Meroplankton accounted for 34.2% of the total SMZ biomass. The majority of this biomass was associated with amphinomid polychaete larvae in samples from July. However, a considerable amount of meroplankton was found from April to September and their relative contribution to SMZ biomass was high in some samples (range: 0%–85%; Figure 2c).

Hierarchical clustering and PCA identified six distinct clusters (Figure 3b). We identified *Oithona* spp. as the only small taxon that contributed to the biomass of all six clusters (Figure 4). Three meroplankton taxa, Cirripedia, Gastropoda, and Bryozoa, contributed to four clusters, while the remaining taxa contributed to three (1 taxon), two (4 taxa), or one (5 taxa) cluster. However, the contribution of Cirripedia, Gastropoda, and Bryozoa was below 2% for one cluster each, and thus, only *Oithona* spp. contributed considerably to more than three clusters. With few exceptions, samples from the same sampling date were assigned to the same cluster (Figure 3b) and, therefore, a significant part of the variation was explained by the sampling date, but not by the sampling station (Figure 3b).

Meroplanktonic taxa dominated the biomass in clusters D and E (i.e., >50% of SMZ biomass) and contributed 13%–16% to the SMZ biomass of clusters B, C, and F (Figures 3b and 4). Cluster A (September) contained only 7% meroplankton and was dominated by five small holoplanktonic copepod taxa. Two species which were virtually absent from all other samples, *Centropages typicus* and *Temora longicornis*, accounted for approximately 45% of the SMZ biomass in cluster A. Similarly, samples from July (cluster E) were characterized by a particularly high biomass of amphinomid polychaetes (55%), which did not contribute to the biomass of any other month. These two clusters, which were dominated by taxa that were exclusively collected on a single sampling date, were the two clusters with the highest SMZ biomass.

Cluster C represents the spring community and contains all but one sample from 1 April and all samples from 1 May. Although *Oithona* spp. and Clausocalanidae were the dominant small taxa in these samples, cluster C was clearly distinguished from all other clusters by abundant cirriped and copepod nauplii (Figures 3b and 4). In April, both types of nauplii were found in similar numbers and accounted for a similar total biomass (Table 3). Regarding their length and individual biomass, cirriped nauplii were smaller than copepod nauplii in early spring. Within a month, both groups increased their total biomass similarly. The median length of cirriped nauplii increased from 392 µm to 703 µm and the individual weight increased by one order of magnitude, while their abundance did not change from April to May. In contrast, copepod nauplii increased in numbers, but individual size stayed very similar. The mean individual weight of the copepod nauplius even decreased slightly from April to May.

**TABLE 3:** Abundance, individual biomass, total biomass, and length for cirriped nauplii and copepod nauplii in samples collected 1. April and 1. May

	1. April 2014			1. May 2014		
	Coast	Bank	Trough	Coast	Bank	Trough
**Abundance [ind./m^2^]**						
Cirriped nauplii	4,128	3,136	0*	4,000	2,880	1,200
Copepod nauplii	1,120	5,248	753*	12,400	30,933	21,688
**Individual biomass [mg DW/ind.]**						
Cirriped nauplii	0.0030	0.0029	–*	0.0208	0.0101	0.0435
Copepod nauplii	0.0054	0.0035	–*	0.0029	0.0027	0.0023
**Total biomass [mg DW/m^2^]**						
Cirriped nauplii	12.496	8.993	–*	83.179	28.963	61.811
Copepod nauplii	6.096	18.593	–*	35.718	83.827	49.959
**Length [µm]**						
Cirriped nauplii	385 (83)	434 (67)	–	748 (119)	725 (35)	632 (460)
Copepod nauplii	500 (54)	546 (42)	548 (49)	496 (37)	489 (47)	506 (77)

Note

“*” indicates sample with too little material for biomass determination. Abundance estimates for this sample are based on the initial size measurement. Length measurements are presented as means (1 *SD*).

### 3.3 Length–weight relationships

**FIGURE 5:**
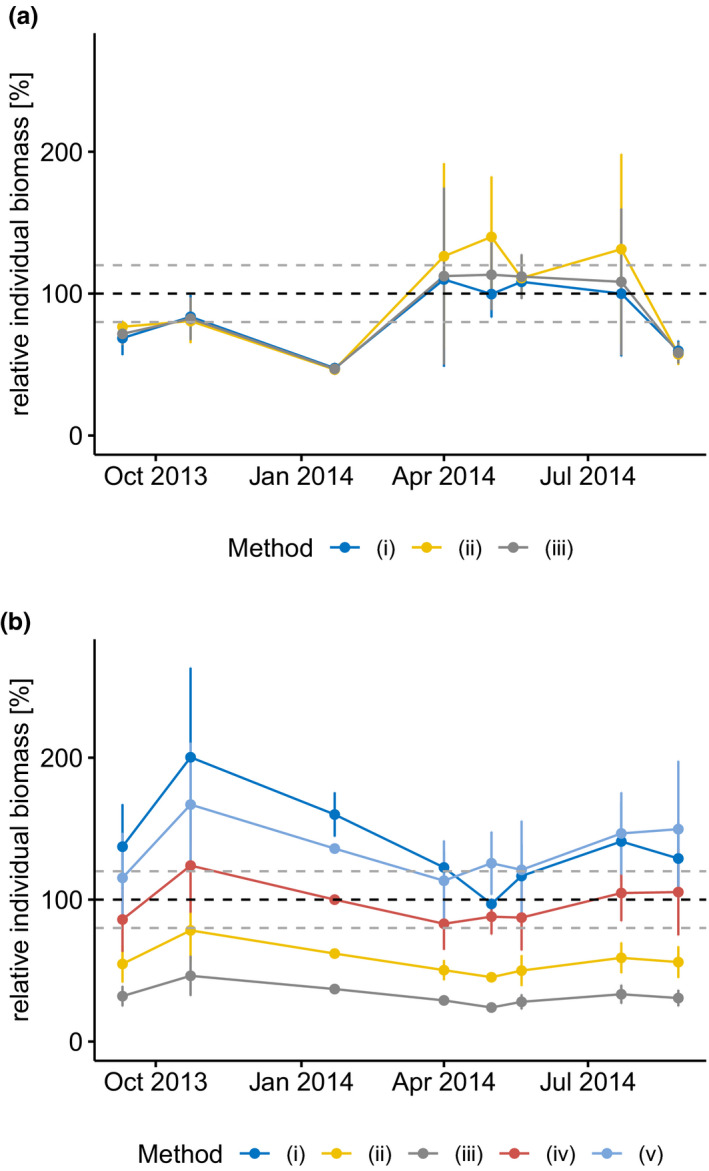
Calculated individual biomass for (a) *Calanus* spp. and (b) *Oithona* spp. Mean (±1 standard deviation) calculated individual biomass of the three sampling stations is given for each sampling event for each method (Biomass calculation methods are given in Table 2). The calculated individual biomass is given relative to the measured mean individual biomass in each sample (100%—black dashed line). Grey dashed lines indicate 80% and 120% of the measured mean individual biomass

Overall, comparisons between calculated (based on published length–weight relationships) and measured average individual biomass of *Calanus* spp. and *Oithona* spp. indicated significant variability and rarely resulted in a satisfactory estimation (±20%) of the measured biomass (Figure 5). For *Calanus* spp., the three employed length–weight relationships all had a strong seasonal bias. The two exponential formulas (method i and iii) reflected the measured biomass well from April to July. The log‐linear model according to Hirche (1991), however, performed more poorly than the exponential formulas during this period and overestimated *Calanus* biomass by more than 20% during 3 (out of 4) sampling events. For the rest of the year, the biomass was consistently underestimated by all methods.

The choice of conversion method for *Oithona* had a huge impact on estimates all year round. For *Oithona* spp., the exponential formula for *O. hebes* according to Ara (2001) performed clearly better than all other methods (Figure 5). The other length–weight relationships either consistently overestimated or consistently underestimated the biomass. The application of a fixed individual weight of 3 µg performed well during spring (April–May) but lead to a strong overestimation of the *Oithona* spp. biomass in autumn and winter.

## 4 DISCUSSION

While our study confirmed *Calanus* spp. as the dominant mesozooplankton in the Lofoten–Vesterålen region, our application of length–weight relationships demonstrates that our knowledge about *Calanus* biomass is seasonally biased and strongly underestimates the standing stock during autumn and winter. Furthermore, we identified the pteropod *L. retroversa* as an important contributor to the mesozooplankton biomass in autumn. The focus on SMZ indicates a complex seasonal succession that includes both holoplankton and meroplankton and is hidden behind the dominant *Calanus* biomass. Low total SMZ biomass in early April indicates that the entire SMZ community plays a potentially important role as prey for larval fish on the Lofoten–Vesterålen shelf, which are abundant at least from late March (herring) to mid‐May (cod) (Fossum & Ellertsen, 1994; Fossum & Moksness, 1993). Furthermore, our results identified particularly high biomass of amphinomid polychaete larvae in July. This high amphinomid biomass can potentially represent an unexplored link to higher trophic levels in the pelagic zone or a vector of carbon export to the benthos.

### 4.1 Large mesozooplankton

We found an overall dominance of *Calanus* spp. in the Lofoten–Vesterålen region that was comparable to other studies of zooplankton biomass in high latitude systems (Arashkevich et al., 2002; Blachowiak‐Samolyk et al., 2008) and points toward *Calanus* spp. as the key taxon supporting the large stocks of marine mammals, sea birds, and adult fish, and also seems to justify the general focus of ecosystem models on *Calanus* spp. (Renaud et al., 2018; Wassmann et al., 2006). However, 69% of the *Calanus* biomass was collected over the deep trough, indicating that *Calanus* spp. is specifically important over troughs (this study) and further off‐shore regions (Basedow et al., 2019).

Furthermore, our results highlight a particularly high biomass of *Limacina retroversa* at the end of the productive season (July–October). A similar high contribution of mollusks (primarily *L. retroversa*) to total zooplankton biomass is also known from the North Sea where Hay et al. (1991) report high mollusk biomass in October with an additional increase in November/December, before a drop in January. We were not able to collect samples between October and January, and accordingly, we do not know when *L. retroversa* disappeared from the study area. Meinecke and Wefer (1990), however, reported sedimentation of *L. retroversa* shells in the Lofoten basin from August to November with a peak in October, and therefore, we assume that most *L. retroversa* disappeared from the study region shortly after our sampling in the end of October. Hay et al. (1991) pointed out that a considerable part (up to 50%) of mollusk DW represents the shell and accordingly the measured DW should be considered an overestimate of the biomass. Nonetheless, the high biomass of *L. retroversa* from July to October and the extreme dominance of *L. retroversa* in October in the shallow parts of our study region (Figure 4; 81.5% of biomass in cluster 2) indicates a potentially important role as prey species for higher trophic levels at shallow shelf regions in autumn, similar to the importance of the more Arctic species *L. helicina* (Karnovsky et al., 2008) or their Southern Ocean counterparts *L*. *retroversa australis* and *L. rangii* (Hunt et al., 2008).

### 4.2 Methods of plankton biomass estimation

We used direct measurements of dry weights of formalin fixed samples. This approach is tedious and has the shortcoming that rare taxa can only be included at low taxonomic resolution. We were, however, able to describe the seasonal development of the biomass of 17 holoplankton and 9 meroplankton taxa, and 2 developmental stages of ichthyoplankton. Accordingly, our data attained a similar taxonomic resolution for holoplankton and ichthyoplankton as used in previous studies of these community components in our study region (Eiane et al., 2018). The nine meroplanktonic taxa with biomass data, however, were far below the 65 meroplankton taxa known to occur in the same community (Silberberger et al., 2016). This large discrepancy was a result of most meroplankton being rather small or low in abundance, and accordingly, meroplankton had to be aggregated on a higher taxonomic level to ensure that our biomass measurements were not biased against meroplankton. While this is a shortcoming for the taxonomic resolution of meroplankton in comparison to holoplankton in our study, we achieved a similar taxonomic resolution as other studies that used conversion factors for meroplankton biomass estimation (e.g., Coyle & Paul, 1990; Stübner et al., 2016).

Furthermore, it is likely that the weighing of such small organisms involves comparatively high measurement errors, and that the applied correction factor for the formalin fixation is not equally suitable for all taxa. Accordingly, the discrepancy between our measured and calculated weights for *Calanus* and *Oithona* could stem from the use of formalin preserved samples in our study. However, only Uye (1982) clearly states that fresh zooplankton was used in his study. All other studies seem to either have used formalin preserved samples or supplemented literature data based on formalin preserved samples with data from fresh samples. While the zooplankton material is not well described in all the publications, a predominant use of formalin preserved samples is apparent, especially for *Calanus*. Mumm (1991) and Hirche (1991) wrote that they used formalin preserved samples to develop their formulas. Furthermore, Mumm (1991) reports that a formalin weight loss of 38% was assumed for copepods. Accordingly, our data are directly comparable to Mumm (1991). No mentioning of correction for formalin preservation was made in Hirche (1991), and we do not know how the material for the formula in Cohen and Lough (1981) was treated. However, due to the similarity of the calculated weights of *Calanus* across all three methods, we consider it most likely that all formulas were developed on formalin preserved samples with a correction for formalin preservation similar to the 40% weight loss assumed in our study. In contrast, *Oithona* was treated differently to develop the formulas. Uye (1982) used fresh material. Ara (2001) used formalin preserved samples, but did not correct for formalin preservation. Accordingly, the formulas from Uye (1982) and Ara (2001) are not directly comparable to our data. Nonetheless, our analysis showed that the formula for *Oithona hebes* in Ara (2001) can be used to satisfactory estimate year‐round *Oithona* biomass in the Lofoten–Vesterålen region for formalin preserved samples after formalin correction. The widely used individual weight of 0.003 mg per individual in Blachowiak‐Samolyk et al. (2008) is based on an average of three other studies (Hanssen, 1997; Mumm, 1991; Richter, 1994). However, Richter (1994) and Hanssen (1997) refer back to Mumm (1991) and accordingly it is based on formalin preserved samples and above mentioned correction. Since the individual *Oithona* weight of 0.003 mg is applied in virtually all sub‐Arctic studies (e.g., Richter, 1994; Blachowiak‐Samolyk et al., 2008; Stübner et al., 2016) and *Calanus* weight–length relationships were established on formalin preserved samples, we conclude at this point that our understanding of sub‐Arctic mesozooplankton biomass is based on formalin preserved and corrected data. Although this is an observation that is not related to our results, we consider it important to highlight this fact as both reviewers expressed their concern about the use of formalin preserved zooplankton samples for biomass determination and questioned whether they could be compared with weight–length relationships from the literature.

Our application of length–weight relationships for *Calanus* spp. and *Oithona* spp. demonstrated that the commonly used biomass estimation methods for the two most common mesozooplankton taxa in the sub‐Arctic introduce huge seasonal biases. For *Calanus* spp., we found a consistent underestimation of the biomass from late August to January, the period with large copepodite stages (prosome length >2 mm) dominating the samples. From April to July, the period with various size classes present in the same samples, the exponential length–weight relationships according to Cohen and Lough (1981) and Mumm (1991) performed well. However, since the winter samples demonstrated that biomass of large individuals is underestimated with these formulas, we consider it likely that the formulas overestimate the weight of the small copepodite stages. This overestimation of the biomass of small *Calanus* must be even stronger for the log‐linear weight–length relation according to Hirche (1991) as it consistently overestimated the biomass in the spring/summer samples.

Similar to the observations for *Calanus*, we found the commonly applied individual biomass of 3 µg for *Oithona* spp. to perform best during spring (April–May), while the *Oithona* biomass was strongly overestimated throughout the rest of the year. Consequently, we suggest that our knowledge about the mesozooplankton biomass in the sub‐Arctic is most accurate for spring and early summer, while considerable differences between true and calculated biomass for autumn and winter have to be expected.

We applied length–weight relationships only to the two most common taxa in the study region and worldwide. Accordingly, species‐ or genus‐specific length–weight relationships were available for *Calanus* and *Oithona*. Since such taxon‐specific length–weight relationships are not available for most other taxa, we assume that literature‐based conversion factors retrieved from higher taxonomic levels for other taxa are likely more biased. Since the calculated biomass was already strongly biased for *Calanus* and *Oithona*, we argue that an increased effort to measure mesozooplankton biomass is needed. In general, the commonly used approach to use a variety of taxon‐specific conversion factors (Arashkevich et al., 2002; Richter, 1994; Stübner et al., 2016) comes with several drawbacks: (a) Conversion factors for most taxa are based on relatively few studies with relatively few replicates. (b) Conversion factors are not available for all taxa and conversion factors from similar taxa at different locations are used as surrogates. This is especially problematic for meroplankton taxa with a typically low taxonomic resolution, which results often in a single individual dry mass value being applied for a complete class, like Polychaeta or Bivalvia (e.g., Stübner et al., 2016). (c) Conversion factors are not adjusted for season. (d) Conversion factors from different studies are not truly comparable as they result in sometimes very different biomass estimates (our study, Blachowiak‐Samolyk et al., 2008). (e) Taxonomic uncertainties with many planktonic species, even for the dominant *Calanus* spp. (Choquet et al., 2018), may affect biomass estimates based on conversion factors.

In our study area, a dominance of *C. finmarchicus* can be assumed (Choquet et al., 2017). However, *C. glacialis* and even *C. hyperboreus* or *C. helgolandicus* can be advected onto the northern Norwegian shelf from fjords and the northern North Sea (Choquet et al., 2017). Since these species cannot be separated according to prosome length in our study region (Choquet et al., 2018), we do not know which species contributed to the *Calanus* biomass in our study. Nonetheless, this did not lead to problems with the application of length–weight relationships. Hirche (1991) and Mumm (1991) developed their length–weight relationship for a mix of *C. finmarchicus* and *C. glacialis*. The formula from Cohen and Lough (1981) was developed for *C. finmarchicus*. However, species identification in these studies was done morphologically and accordingly our *Calanus* would have been identified as *C. finmarchicus* when the same classification would be applied.

### 4.3 Potential ecological functions of small mesozooplankton

#### 4.3.1 *Prey* *for larval fish*

Spring is the period when first‐feeding fish larvae are abundant (Fossum & Ellertsen, 1994; Fossum & Moksness, 1993) over the Lofoten–Vesterålen shelf. Although our sampling method did not target ichthyoplankton, the presence of cod eggs and larvae in the plankton during April and May 2014 (Appendix S1, Eiane et al., 2018) supports the assumption that the spring SMZ community represents potential prey items available for fish larvae. Knowledge about the prey composition of fish larvae of species spawning in sub‐Arctic Norway is largely limited to some early studies that found copepod nauplii dominating the gut content of larval herring and cod (Bjørke, 1978; Fossum & Ellertsen, 1994; Tilseth, 1984). On this basis, more recent studies often limited their efforts to the identification of a spatio‐temporal overlap between fish larvae and *Calanus* nauplii as key prey and therefore as a basis for estimating/predicting larval success (Espinasse et al., 2016, 2017; Sundby, 2000). The low total SMZ biomass in early April (Figure 2), however, indicates that fish larvae cannot afford selective feeding in early spring and that the entire SMZ community could be important prey items for fish larvae. A strong increase of the total SMZ biomass from early April to May indicates that a possible oversupply of suitable food in early May might allow for selective feeding. Studies that found *Calanus* nauplii dominating in stomachs of first‐feeding cod larvae were typically conducted in late April or May (Fossum & Ellertsen, 1994).

Mesocosm experiments have shown that first feeding cod larvae generally select for copepod nauplii, but prey opportunistically on other less nutritious small taxa if copepod nauplii with a suitable size are low in abundance (van der Meeren & Næss, 1993). Overall, prey size tends to be the primary determinant of diets of first feeding fish larvae (Blaxter, 1963; Fossum & Ellertsen, 1994; Kane, 1984). For example, cod is limited to prey sizes of 120–400 µm for the first 30 days posthatching, after which the size range of prey increases to 200–2000 µm (Fossum & Ellertsen, 1994). We assume, therefore, that the entire spring SMZ biomass (and also a part of the larger size fraction) in our study is suitable prey for older cod larvae (>30 days posthatching). Due to the overall low mesozooplankton biomass in April, we suggest that cod larvae that hatched in February and early March are likely to feed nonselectively on the entire SMZ biomass. Larvae that hatched during March, however, will be limited to prey that is smaller than 400 µm. We measured the length of a total of 136 individuals smaller than 400 µm for early April, and the majority of these individuals belonged to three taxa/groups: ophiopluteus larvae (46%), cirripedia nauplii (26%), and *Fritillaria borealis* (20%). Due to their low weight, however, DW data of Ophiopluteus larvae were considered unreliable (*i.e*., weight gain less than 4 × SD of measurement error) and excluded from the analysis. A single *Calanus* nauplius smaller than 400 µm was measured in April (392 µm), and only one additional *Calanus* nauplius had a length between 400 and 450 µm.

The copepod nauplii in our study had an average size of approximately 500 µm (Table 3), which indicates that these nauplii most likely represent *Calanus* nauplii (stages N4–N6) throughout the spring (Campbell et al., 2001; Hygum et al., 2000). One could argue that this absence of early *Calanus* nauplii is most likely related to the sampling efficiency of the chosen mesh size. The sampling efficiency of a 200‐µm mesh has to be considered nonquantitative for such small taxa and abundances of taxa below 500 µm are likely underestimated by 90% or more in our samples (Riccardi, 2010). Indeed, we assume that our sampling was biased toward the collection of specific small taxa that are more likely to get stuck in the mesh with their appendages (like ophiopluteus larvae, cirripedia nauplii, and *Fritillaria borealis*). However, in contrast to the earlier naupliar stages of *Calanus*, other taxa with no appendages were collected in samples from late spring to autumn. The bivalve veliger larvae we measured as part of this study were on average 328 µm long, and 23% of the gastropod veliger were smaller than 300 µm. We consider this as an indication that some smaller *Calanus* nauplii should have been caught in our net (although not quantitatively) if they were present in high numbers. However, only 6% of the measured *Calanus* nauplii in our study were smaller than 400 µm, with the shortest individual being 329 µm. *Calanus* naupliar stage 3 is shorter than 300 µm (Hygum et al., 2000), and accordingly, we only measured stages N4–N6. Based on this consideration, we suggest that the complete lack of earlier naupliar stages of *Calanus* in our samples suggests that they were not abundant in our study region. This absence could reflect the distance of our study region to the *Calanus* overwintering population in the inner part of Vestfjorden (shortest distance: ~75 nm; distance along main dispersal pathway: ~150 nm) and the time it takes for the *Calanus* eggs and nauplii to be advected to the outside of the Lofoten and Vesterålen islands (over 20 days) (Espinasse et al., 2016, 2017; Silberberger et al., 2016). Advection of nauplii from offshelf waters is low in most years (Espinasse et al., 2017) and particle‐tracking suggests a shelf origin of spring nauplii in 2014, when our samples were collected (Silberberger et al., 2016). Although this is speculation, we suggest that other prey than *Calanus* nauplii may be important alternative prey for first‐feeding cod larvae in early spring in our study region.

Similarly, the early spring community also represents the available prey for first‐feeding larvae of Norwegian spring spawning herring during March and the first days of April (Fossum & Moksness, 1993). Fossum and Moksness (1993) reported a mismatch between first‐feeding herring and their assumed prey (copepod nauplii and eggs) that displayed a low abundance throughout the first half of April. Herring larvae, however, can survive for up to three weeks when fed with early naupliar stages of Cirripedia (Dempsey, 1978). Furthermore, herring larvae show increased activity when presented with washings and extracts of *Semibalanus balanoides* (Dempsey, 1978), the dominant cirriped in the plankton community in the Lofoten–Vesterålen region in early April (Silberberger et al., 2016), even before they started feeding. Based on this suitability of Cirripedia nauplii as a prey for first‐feeding herring larvae and their high contribution to the total mesozooplankton biomass at the coast in early spring (17% or 19% when ichthyoplankton is excluded), we suggest that Cirripedia can act as an alternative prey item for first‐feeding herring larvae that drift close to the coast. Herring larvae that drift further offshore, however, seem more likely to rely on small sized copepods, copepod nauplii, and other small‐sized holoplankton like *F. borealis*.

#### 4.3.2 Meroplankton as a vector of vertical carbon flux

We encountered high meroplankton biomass (up to 1,084 mg DW/m^2^) during July, which contributed with 12.7%, 9.8%, and 1.7% to the total mesozooplankton biomass close to the coast, over the shallow bank, and over the deep trough, respectively (Figure 2c). The majority of this biomass was accounted for by larvae of amphinomid polychaetes (490–885 mg DW/m^2^). This high contribution of benthic polychaete larvae at the coast and over the shallow bank in summer is comparable to reports of Hickel (1975) from the Wadden Sea of Sylt, where he found polychaete larvae to contribute on average 15% of the summer zooplankton biomass. Pelagic larvae of benthic invertebrates have subsequently been confirmed as an important component of the summer zooplankton community in the North Sea (Franco‐Santos et al., 2019; Kirby et al., 2007, 2008).

We suggest that the high summer meroplankton biomass in our study indicates a similar importance of meroplankton in the Lofoten–Vesterålen region during summer. Benthic macrofauna communities on the Lofoten–Vesterålen shelf have an average abundance of 1,047 individuals per m^2^ (Silberberger et al., 2019). This is considerably lower than the average of over 50,000 meroplankton individuals per m^2^ surface area on 22 July 2014 (Silberberger et al., 2016). Furthermore, amphinomid polychaetes are not abundant on the Lofoten–Vesterålen shelf (Silberberger et al., 2019). Accordingly, the vast majority of the summer meroplankton biomass must have a different fate than successful recruitment. A similar mismatch between meroplankton and adult populations is known from the Chukchi Sea (Ershova et al., 2019). Ershova et al. (2019) concluded that this means that the meroplankton is consumed either by pelagic or benthic predators. Lalande et al. (2020) found that daily sinking rates of polychaete larvae in the Chukchi Sea exceed 10,000 individuals per m^2^ in the second half of September. Similarly, we consider it likely that the majority of summer meroplankton reaches the sediment, since the primary summer feeding grounds of herring, mackerel, and blue whiting are located further offshore (Bachiller et al., 2016). The carbon demand of epibenthic communities (the potential consumers of settling larvae) in our study region has not been assessed. However, assuming a total epibenthic carbon demand in the range of 6 to 70 g C m^−2^ y^−1^ as found on Svalbardbanken in the Barents Sea (Kędra et al., 2013), the average 278.6 mg C m^−2^ associated with the settlement of all meroplankton in our samples from July could account for 0.4 to 5% of the epibenthic annual carbon demand. While this contribution might seem small, we consider it a better food source for benthic carnivores and omnivores than other forms of organic matter that reach the seafloor. Microalgae, zooplankton fecal pellets, and detritus first have to be consumed by primary consumers, while settled meroplankton is directly available to benthic carnivores and omnivores that are typical for our study region (Silberberger et al., 2018).

We suggest that this rapid (relative to sinking phytodetritus) and active transport of pelagically derived carbon directly to the benthic zone could potentially become more important in Arctic and sub‐Arctic regions in the future. In a warmer future Arctic/sub‐Arctic, it is likely that meroplankton biomass will increase due to northward range expansion of southern species (Narayanaswamy et al., 2010), whose life history involves planktotrophic larvae more frequently than that of Arctic species (Clarke, 1992; Thorson, 1950). This may, then, lead to an increased carbon export *via* settling meroplankton with potential consequences for trophic interactions of the whole ecosystem. Whereas this prediction is largely speculative at this point, ignoring a potentially important vector like meroplankton may hinder a better understanding of ecosystem functioning of high latitude ecosystems.

## 5 CONCLUSION

Our study confirmed the dominance of *Calanus* spp. in the mesozooplankton biomass and an overall rather small contribution of meroplankton to this biomass on an annual basis. Nonetheless, we found meroplankton representing a considerable fraction of the SMZ biomass in early April (prior to the *Calanus* nauplii peak) and a meroplankton biomass peak in July contributing approximately 10% of the total net caught mesozooplankton biomass at the coast and over the shallow part of the shelf.

We suggest that the complex succession of SMZ (holoplankton and meroplankton) indicates recruitment of the large boreo‐arctic fish stocks may be less tightly coupled to the early life cycle of *Calanus* spp. than previously assumed, as they may utilize small holoplanktonic and meroplanktonic taxa as an alternative source of food throughout the spring and early summer. This could be advantageous in the context of the ongoing climate change, since variable changes in phenology of different components of pelagic food webs are expected to result in increased trophic mismatch situations and reduced recruitment success of fish in the future (Rijnsdorp et al., 2009). In general, the availability of multiple SMZ taxa originating from two different ecosystem components (plankton–benthos) with relatively high biomass ensures a longer time‐window with suitable prey for first‐feeding fish larvae over the northern Norwegian continental shelf and might reduce the risk of mismatch situations.

High abundances in meroplankton biomass can comprise an important and virtually unstudied bidirectional vector of carbon transport between pelagic zone and benthos. On the one hand, a potential importance of meroplankton in fish diets suggests shunting of benthic carbon to the pelagic food web. On the other hand, settling meroplankton may contribute significantly to vertical flux during summer (the time when benthic carbon demand is highest) and provide a high‐quality food source for the benthos. Overall, our findings indicate that roles of functionally different components of the mesozooplankton community need to be better described and quantified. Specifically, we suggest that benthic populations as the source of meroplanktonic larvae should receive more attention to fully understand the functioning of pelagic systems.

We recommend the increased use of direct mesozooplankton biomass measurement in future studies when possible, since the currently applied conversion factors are highly biased, and only allow for incomplete understanding of ecosystem processes and prediction of ecosystem‐wide responses to ongoing climate change.

## CONFLICT OF INTEREST

The authors declare no conflicts of interest.

## AUTHOR CONTRIBUTION

**Marc J. Silberberger:** Conceptualization (equal); Data curation (lead); Formal analysis (lead); Investigation (lead); Methodology (lead); Visualization (lead); Writing‐original draft (lead); Writing‐review & editing (lead). **Paul E. Renaud:** Conceptualization (equal); Formal analysis (supporting); Funding acquisition (equal); Investigation (equal); Methodology (supporting); Writing‐original draft (supporting); Writing‐review & editing (equal). **Ketil Eiane:** Conceptualization (equal); Formal analysis (supporting); Funding acquisition (equal); Investigation (equal); Methodology (supporting); Writing‐original draft (supporting); Writing‐review & editing (equal). **Henning Reiss:** Conceptualization (equal); Formal analysis (supporting); Funding acquisition (equal); Investigation (equal); Methodology (supporting); Writing‐original draft (supporting); Writing‐review & editing (equal).

## Supporting information

Appendix S1

## Data Availability

Mesozooplankton biomass and length measurements can be accessed on DataverseNO: https://doi.org/10.18710/07JT6E
