# Supplementary material for: Seasonal dynamics of mesozooplankton biomass over a sub‐Arctic continental shelf
Source: Ecol Evol. 2021 May 25;11(13):8713–29. doi: 10.1002/ece3.7681 (PMC8258191; doi:10.1002/ece3.7681)
Supplement: Supplementary file 1 — Appendix S1 [file ECE3-11-8713-s001.docx]

**Supplement 1.** Zooplankton dry weight, organic carbon, and energy content of all samples. Absolute values are given for the total zooplankton. Absolute values and contribution to the total zooplankton (in %) is given for holoplankton (Holo), meroplankton (Mero), and ichthyoplankton (Ichthyo).

| Sample | Dry weight [mgDW/m^2^] | | | |  | Organic carbon [mgC_org_/m^2^] | | | |  | Energetic content [J/m^2^] | | | |
| --- | --- | --- | --- | --- | --- | --- | --- | --- | --- | --- | --- | --- | --- | --- |
|  | Total | Holo | Mero | Ichthyo |  | Total | Holo | Mero | Ichthyo |  | Total | Holo | Mero | Ichthyo |
| **10.09.13** |  |  |  |  |  |  |  |  |  |  |  |  |  |  |
| Coast | 3497.0 | 3364.4 (96.2%) | 132.6 (3.8%) | 0 (0%) |  | 1584.5 | 1537.8 (97.1%) | 46.7 (2.9%) | 0 (0%) |  | 77614 | 75513 (97.3%) | 2101 (2.7%) | 0 (0%) |
| Bank | 4860.1 | 4860.1 (100%) | 0 (0%) | 0 (0%) |  | 2385.5 | 2385.5 (100%) | 0 (0%) | 0 (0%) |  | 116912 | 116912 (100%) | 0 (0%) | 0 (0%) |
| Trough | 3652.5 | 3652.5 (100%) | 0 (0%) | 0 (0%) |  | 1656.7 | 1656.7 (100%) | 0 (0%) | 0 (0%) |  | 82645 | 82645 (100%) | 0 (0%) | 0 (0%) |
| **23.10.13** |  |  |  |  |  |  |  |  |  |  |  |  |  |  |
| Coast | 1844.8 | 1844.8 (100%) | 0 (0%) | 0 (0%) |  | 673.4 | 673.4 (100%) | 0 (0%) | 0 (0%) |  | 32933 | 32933 (100%) | 0 (0%) | 0 (0%) |
| Bank | 778.9 | 778.9 (100%) | 0 (0%) | 0 (0%) |  | 279.2 | 279.2 (100%) | 0 (0%) | 0 (0%) |  | 13617 | 13617 (100%) | 0 (0%) | 0 (0%) |
| Trough | 3436.2 | 3436.2 (100%) | 0 (0%) | 0 (0%) |  | 1544.6 | 1544.6 (100%) | 0 (0%) | 0 (0%) |  | 80282 | 80282 (100%) | 0 (0%) | 0 (0%) |
| **22.01.14** |  |  |  |  |  |  |  |  |  |  |  |  |  |  |
| Coast | 24.6 | 24.6 (100%) | 0 (0%) | 0 (0%) |  | 12.4 | 12.4 (100%) | 0 (0%) | 0 (0%) |  | 638 | 638 (100%) | 0 (0%) | 0 (0%) |
| Bank | 92.0 | 92.0 (100%) | 0 (0%) | 0 (0%) |  | 43.9 | 43.9 (100%) | 0 (0%) | 0 (0%) |  | 2280 | 2280 (100%) | 0 (0%) | 0 (0%) |
| Trough | 298.1 | 298.1 (100%) | 0 (0%) | 0 (0%) |  | 146.3 | 146.3 (100%) | 0 (0%) | 0 (0%) |  | 7739 | 7739 (100%) | 0 (0%) | 0 (0%) |
| **01.04.14** |  |  |  |  |  |  |  |  |  |  |  |  |  |  |
| Coast | 73.6 | 52.9 (71.9%) | 12.5 (17.0%) | 8.2 (11.1%) |  | 34.9 | 25.9 (74.2%) | 5.5 (15.8%) | 3.6 (10.3%) |  | 1644 | 1242 (75.5%) | 213 (13.0%) | 188 (11.4%) |
| Bank | 525.6 | 470.9 (89.6%) | 13.7 (2.6%) | 41.0 (7.8%) |  | 257.5 | 234.1 (90.9%) | 5.6 (2.2%) | 17.7 (6.9%) |  | 13308 | 12157 (91.4%) | 212 (1.6%) | 939 (7.1%) |
| Trough | 265.7 | 246.3 (92.7%) | 0 (0%) | 19.4 (7.3%) |  | 134.4 | 126.0 (93.8%) | 0 (0%) | 8.4 (6.3%) |  | 6536 | 6093 (93.2%) | 0 (0%) | 443 (6.8%) |
| **01.05.14** |  |  |  |  |  |  |  |  |  |  |  |  |  |  |
| Coast | 3036.0 | 2656.3 (87.5%) | 95.6 (3.1%) | 284.1 (9.4%) |  | 1480.3 | 1319.5 (89.1%) | 38.1 (2.6%) | 122.7 (8.3) |  | 75900 | 67907 (89.5%) | 1487 (2.0%) | 6507 (8.6%) |
| Bank | 9817.2 | 9485.2 (96.6%) | 29.0 (0.3%) | 303.0 (3.1%) |  | 4846.6 | 4703.1 (97.0%) | 12.7 (0.3%) | 130.9 (2.7%) |  | 249543 | 242110 (97.0%) | 494 (0.2%) | 6939 (2.8%) |
| Trough | 23216.3 | 22595.8 (97.3%) | 61.8 (0.3%) | 558.8 (2.4%) |  | 11543.2 | 11274.8 (97.7%) | 27.0 (0.2%) | 241.4 (2.1%) |  | 598027 | 584176 (97.7%) | 1055 (0.2%) | 12796 (2.1%) |
| **20.05.14** |  |  |  |  |  |  |  |  |  |  |  |  |  |  |
| Coast | 1485.8 | 1428.6 (96.2%) | 36.7 (2.5%) | 20.5 (1.4%) |  | 728.0 | 706.4 (97.0%) | 12.8 (1.8%) | 8.8 (1.2%) |  | 38694 | 37803 (97.7%) | 422 (1.1%) | 469 (1.2%) |
| Bank | 15494.4 | 14523.0 (93.7%) | 248.6 (1.6%) | 722.9 (4.7%) |  | 7559.1 | 7187.1 (95.1%) | 59.7 (0.8%) | 312.3 (4.1%) |  | 395773 | 376336 (95.1%) | 2884 (0.7%) | 16554 (4.2%) |
| Trough | 13303.6 | 13303.6 (100%) | 0 (0%) | 0 (0%) |  | 6620.2 | 6620.2 (100%) | 0 (0%) | 0 (0%) |  | 334783 | 334783 (100%) | 0 (0%) | 0 (0%) |
| **22.07.14** |  |  |  |  |  |  |  |  |  |  |  |  |  |  |
| Coast | 8529.8 | 7445.9 (87.3%) | 1083.9 (12.7%) | 0 (0%) |  | 3914.4 | 3510.9 (89.7%) | 403.5 (10.3%) | 0 (0%) |  | 189805 | 170831 (90.0%) | 18974 (10.0%) | 0 (0%) |
| Bank | 6894.4 | 6220.8 (90.2%) | 673.6 (9.8%) | 0 (0%) |  | 2977.5 | 2728.4 (91.6%) | 249.1 (8.4%) | 0 (0%) |  | 147045 | 135020 (91.8%) | 12025 (8.2%) | 0 (0%) |
| Trough | 28507.0 | 28015.6 (98.3%) | 491.5 (1.7%) | 0 (0%) |  | 14116.0 | 13932.7 (98.7%) | 183.3 (1.3%) | 0 (0%) |  | 742000 | 733251 (98.8%) | 8748 (1.2%) | 0 (0%) |
| **27.08.14** |  |  |  |  |  |  |  |  |  |  |  |  |  |  |
| Coast | 1886.6 | 1859.3 (98.6%) | 27.3 (1.4%) | 0 (0%) |  | 852.3 | 842.4 (98.8%) | 9.8 (1.1%) | 0 (0%) |  | 42787 | 42449 (99.2%) | 338 (0.8%) | 0 (0%) |
| Bank | 2465.4 | 2431.6 (98.6%) | 33.8 (1.4%) | 0 (0%) |  | 1204.1 | 1192.0 (99.0%) | 12.1 (1.0%) | 0 (0%) |  | 61809 | 61356 (99.3%) | 453 (0.7%) | 0 (0%) |
| Trough | 38692.8 | 38635.7 (99.9%) | 57.2 (0.1%) | 0 (0%) |  | 18963.6 | 18940.7 (99.9%) | 23.0 (1.2%) | 0 (0%) |  | 1003811 | 1003312 (>99.9%) | 498 (<0.1%) | 0 (0%) |
